# Editorial: Neural mechanism and effect of acupuncture for central nervous system diseases

**DOI:** 10.3389/fnins.2023.1337612

**Published:** 2024-01-08

**Authors:** Qinhong Zhang, Cunzhi Liu, Xianghong Jing, Hao Chi, Xiaoling Li, Jinhuan Yue, Weixing Pan, Guanhu Yang

**Affiliations:** ^1^Department of Acupuncture and Moxibustion, Heilongjiang University of Chinese Medicine, Harbin, China; ^2^School of Acupuncture-Moxibustion and Tuina, Beijing University of Chinese Medicine, Beijing, China; ^3^Institute of Acupuncture and Moxibustion, China Academy of Chinese Medical Sciences, Beijing, China; ^4^Clinical Medical College, Southwest Medical University, Luzhou, China; ^5^Division of CT and MRI, First Affiliated Hospital of Heilongjiang University of Chinese Medicine, Harbin, China; ^6^Department of Acupuncture and Moxibustion, Vitality University, Hayward, CA, United States; ^7^Janelia Research Campus, Howard Hughes Medical Institute, Ashburn, VA, United States; ^8^Department of Specialty Medicine, Ohio University, Athens, OH, United States

**Keywords:** acupuncture, central nervous system disease, stroke, Parkinson's disease, Alzheimer's disease, migraine, depression, neural mechanism

Central nervous system (CNS) diseases encompass a wide range of disorders that affect the brain and spinal cord (Rothwell et al., 2021). These diseases involve complex neural mechanisms, which refer to the intricate workings and interactions of the neurons and other cells in the CNS (Fontes et al., 2016). The CNS controls and coordinates most of the body's functions, including movement, sensation, cognition, and regulation of vital organs. Thus, any disruption in its functioning can have profound consequences. CNS diseases not only pose a significant burden on the affected individuals but also have a considerable impact on their quality of life. Researchers and healthcare professionals strive to better understand the underlying neural mechanisms involved in these diseases, enabling the exploration of novel therapeutic approaches and targeted interventions that can help manage symptoms and enhance patient outcomes.

As for the treatment modality, acupuncture has been used as a therapeutic practice in China and various other Asian countries for over 2,000 years (Bonica, 1974). Over the years, numerous studies have been conducted to explore the benefits of acupuncture in protecting and managing nerve tissue from injuries, particularly for patients suffering from CNS diseases (Chen et al., 2014; Scheffold et al., 2015; Xiao et al., 2018). The results of these studies have consistently shown that acupuncture exhibits very few adverse effects, making it a safe option for patients with CNS diseases. However, despite the increasing attention and research dedicated to acupuncture's neural mechanism and effects, there is still much that remains unexplored. In order to shed further light on the neural mechanism and effect of acupuncture in the treatment of CNS diseases, we launched a Research Topic about “*Neural mechanism and effect of acupuncture for central nervous system diseases*” on October 24, 2022, inviting researchers to contribute their studies, which help understand the intricate neural mechanisms and therapeutic effects of acupuncture in managing CNS diseases. It is also hoped that this Research Topic expands our knowledge in this field and pave the way for more effective and targeted acupuncture treatments.

The Research Topic “*Neural mechanism and effect of acupuncture for central nervous system diseases*,” published in Frontiers in Neuroscience, featured 22 articles involving 164 authors from seven countries, presenting significant contributions to our understanding of acupuncture's neural mechanism and effect for CNS diseases. These articles can be summarized into two types: neural mechanism of acupuncture and effect of acupuncture (Table 1).

**Table 1:** Summary of included literature features of acupuncture for CNS.

**Study**	**Literature features**
**Neural mechanism of acupuncture on CNS**	
Zhang et al.	Acupuncture alleviates neuroinflammation by inhibiting the activation of the NLRP3 inflammasome pathway
Liu J.-p. et al.	EA and MA have beneficial effects in reducing hypertension and improving cognitive function
Chen et al.	Acupuncture modulates FC between the LC and IFG, insular gyrus, and SMG
Lu et al.	Acupuncture is found to promote motor recovery after stroke by enhancing overall brain connectivity and optimizing functional interactions
Jiang, Deng et al.	Acupuncture is an effective therapeutic approach with a profound impact on the intestinal microbiota and mental health outcomes
Wang J.-X. et al.	New insights into the use of acupuncture for managing poststroke spasticity
Li Y.-Y. et al.	Acupuncture manipulations activated the corresponding brain regions involving in the anti-hypertensive effect
Yue et al.	Comprehensive investigation to gain insights into the current status, hotspots and trends in MRI of WM in AD
Yang et al.	Current research status of acupuncture for MCI
Jin et al.	Impact of scalp acupuncture on CBF and its role in improving symptoms related to brain disorders
Lee and kim	Acupuncture has potential to regulate neurological processes associated with sleep
Fu and Wang	Acupuncture may promote neuroprotection and enhance recovery after stroke
**Effect of acupuncture on CNS**	
Lei et al.	Acupuncture with a specific dose is associated with better therapeutic effects
Li Y.-J. et al.	Acupuncture manipulations can effectively lower blood pressure
Jiang, Zhang et al.	A case report shows that acupuncture may benefit neurological sequelae of electric shock
Fernández-Hernando et al.	Non-invasive techniques showed some positive effects on the treatment of migraines
Pu et al.	Acupuncture and traditional Chinese medical herbs may benefit for TD in children
Li L. et al.	Acupuncture has a positive influence on the short-term recovery of consciousness and long-term outcomes in acute phrase of ICH
Liu Y. et al.	Fire needle acupuncture has positive effects and significant benefits in managing autoimmune disorder
Wang Y. et al.	Cheek acupuncture has an immediate analgesic effect on severe neuralgia associated with peripheral nerve compression or permanent damage/dysfunction of CNS
Shi et al.	Acupuncture benefits episodic migraine for at least three months after the completion of the treatment

## Neural mechanism of acupuncture on CNS

### Acupuncture alleviates neuroinflammation by inhibiting activation of NLRP3 inflammasome

The research conducted by Zhang et al. has found that the activation of NLRP3 inflammasome plays a significant role in the development of CNS. Specifically, the activation of NLRP3 inflammasome exacerbates neuroinflammation and contributes to the progression of CNS diseases. However, the study also suggests that inhibiting the activation of NLRP3 inflammasome could potentially be a key therapeutic target for the treatment of CNS diseases. Furthermore, the research findings indicate that acupuncture may have a beneficial effect on CNS diseases by alleviating neuroinflammation. The mechanism behind this is attributed to the inhibition of the NLRP3 inflammasome pathway by acupuncture. By suppressing the activation of NLRP3 inflammasome, acupuncture interventions demonstrate the potential to improve the progression of CNS diseases by reducing neuroinflammation.

### Acupuncture for hypertension reduction and cognitive function improvement

According to the study conducted by Liu J.-p. et al.  electroacupuncture (EA) and manual acupuncture (MA) have been found to have beneficial effects in reducing hypertension and improving cognitive function. These acupuncture techniques were shown to enhance the functional activities in the specific brain regions associated with these conditions. Moreover, the study found that EA treatment resulted in greater activation of additional brain regions and improved functional connectivity compared to MA treatment.

### Potential mechanism of acupuncture for insomnia

According to Chen et al.'s research, they reported that acupuncture has the ability to regulate the functional connectivity between the locus coeruleus and various regions within the brain such as the inferior frontal gyrus, insular gyrus, and supramarginal gyrus. This finding suggests that acupuncture may have a potential mechanism for the treatment of insomnia. This modulation of functional connectivity (FC) implies that acupuncture could influence the neural pathways and circuits involved in sleep regulation, leading to improvements in sleep quality and duration. By targeting specific brain regions associated with sleep and wakefulness, acupuncture may help restore the balance and proper functioning of the sleep-wake cycle, thereby offering a potential therapeutic approach for individuals suffering from insomnia.

### Neuroimaging mechanisms of acupuncture for motor recovery after stroke

Lu et al. conducted a study where they utilized machine learning to predict the classification of minimal clinically important differences (MCID) for motor improvement after stroke. They aimed to explore the mechanisms underlying brain functional reorganization and the effects of acupuncture on motor recovery. The researchers found that machine learning algorithms were able to identify specific regions of interest (ROIs) in the brain that could accurately predict the extent of motor improvement in stroke patients. Furthermore, they observed that the FC between these ROIs and other brain regions was significantly decreased in stroke patients. The study also revealed that acupuncture treatment could effectively modulate the bilateral cerebral hemispheres and restore abnormal FC in stroke patients. This modulation occurred via different targets within the brain. As a result, acupuncture was found to promote motor recovery after stroke by enhancing overall brain connectivity and optimizing functional interactions.

### Acupuncture for alterations in gut microbiota

In their review article, Jiang, Deng et al. extensively analyzed the studies that investigated the alterations in the gut microbiota following acupuncture therapy. They meticulously examined and addressed the current challenges and potential advancements in the fields of acupuncture, microbiome, and poststroke depression. Through their comprehensive analysis, Jiang, Deng et al. intended to create a foundation for future investigations, facilitating the advancement of acupuncture as an effective therapeutic approach with a profound impact on the intestinal microbiota and mental health outcomes.

### New insights of acupuncture for poststroke spasticity

Based on current evidence from both clinical studies and laboratory research, Wang J.-X. et al. explored new insights into the use of acupuncture for managing poststroke spasticity (PSS), with a particular focus on the antispastic needling technique. They suggest that this technique may have potential effects on both CNS modulations and peripheral adjustments, which could contribute to the management of PSS. The authors also highlight the need for further research in several areas. First, they emphasize the importance of determining the optimal timing and duration of acupuncture intervention for PSS management. They believe that a better understanding of the ideal treatment course can lead to improved outcomes for stroke patients. Additionally, the authors call for more research into the specific application of acupuncture techniques for PSS. They suggest that different needling methods and manipulation techniques may produce varying effects on spasticity, and exploring these possibilities could contribute to the optimization of the antispastic acupuncture regimen. Furthermore, the authors propose investigating different acupoint selection strategies for PSS treatment. They believe that identifying the most effective acupoints for targeting spasticity can enhance the overall efficacy of acupuncture therapy in stroke patients. The authors also emphasize the importance of identifying predictive and aggravating factors of PSS. They argue that a better understanding of these factors can help tailor acupuncture interventions to individual patients and improve treatment outcomes. Lastly, the authors highlight the need for more rigorous study designs and valid objective assessments for spasticity in future research on acupuncture for PSS. They suggest that these improvements can enhance the reliability and validity of study findings and contribute to the overall advancement of acupuncture as a treatment modality for PSS.

### Acupuncture manipulations on blood pressure and brain function

In their study, Li Y.-Y. et al. conducted experiments on spontaneously hypertensive rats to observe the effects of acupuncture manipulations on blood pressure and brain function. They aimed to elucidate the central mechanism behind the anti-hypertensive effect of these manipulations. The researchers found that acupuncture manipulations were able to achieve a hypotensive effect in the spontaneously hypertensive rats. Specifically, they noted that the twirling reducing manipulation had a significantly better hypotensive effect compared to the twirling uniform reinforcing-reducing and twirling reinforcing manipulations. To understand the central mechanism behind these effects, the researchers investigated the activation of brain regions associated with blood pressure regulation and the functional connections between them. They found that the twirling reinforcing and reducing manipulation activated these brain regions, suggesting its involvement in the anti-hypertensive effect. Interestingly, the study also revealed that brain regions involved in motor control, cognition, and hearing were activated during the acupuncture manipulations. This observation led the researchers to hypothesize that the activation of these brain regions may have additional benefits in preventing or mitigating the onset and progression of hypertensive brain damage.

### Current status, hotspots and trends on magnetic resonance imaging of white matter in Alzheimer's disease

Yue et al. conducted a comprehensive investigation to gain insights into the current state of research, key areas of focus, and emerging trends in MRI of WM in patients with AD. Their study extensively reviewed existing publications on the topic, shedding light on the current understanding of WM abnormalities in AD and highlighting the significant areas of research. The findings of this study not only shed light on the current state of research but also identified the key areas of focus and hotspots within the field of MRI of WM in AD. Furthermore, the study's analysis also revealed the frontier trends in the field of MRI of WM in AD. These emerging trends suggest exciting new possibilities and avenues for future research, offering potential breakthroughs and advancements in the diagnosis, treatment, and understanding of AD. By identifying these frontier areas, this study opens up new possibilities for researchers to explore novel approaches, invent cutting-edge techniques, and develop innovative interventions that could ultimately lead to more effective management and treatment of AD.

### Bibliometric analysis of acupuncture for mild cognitive impairment

The study conducted by Yang et al. utilized bibliometric methods to analyze the current research status of acupuncture in the treatment of MCI in great detail. By exploring the current research hotspots and predicting future trends, the study found that the popularity of acupuncture for MCI is steadily increasing with each passing year. The effectiveness of acupuncture for MCI is further enhanced when combined with cognitive training, as it has been found to significantly improve cognitive function. This study also investigated the role of inflammation in MCI and explored how acupuncture can effectively address this issue.

### Impact of scalp acupuncture on cerebral blood flow

In their study, Jin et al. delved into an innovative approach to understand how scalp acupuncture effectively treats brain diseases. They focused on investigating the impact of scalp acupuncture on CBF and its role in improving symptoms related to brain disorders. Through extensive research and analysis, they discovered that the stimulation of specific scalp acupuncture points, targeted areas, or nerves innervating the scalp resulted in a remarkable increase in CBF.

### Mechanism of acupuncture for sleep disorders

In their study, Lee and Kim conducted an investigation to explore the underlying mechanism of acupuncture's effectiveness in treating sleep disorders using a rodent model. The researchers found that sleep disorders were closely associated to various brain regions and neurotransmitters. They found that acupuncture had the potential to regulate neurological processes associated with sleep, such as the catecholamine and BDNF signaling pathways.

### Acupuncture-induced endogenous neuroprotective mechanisms of poststroke

The study by Fu and Wang provides a comprehensive understanding of the potential acupuncture-induced endogenous neuroprotective mechanisms by critically examining a wide range of experimental evidence on the preventive and therapeutic effects that acupuncture exerts on cerebral ischemic stroke. By analyzing various studies and experimental findings, this review sheds light on the specific mechanisms through which acupuncture may promote neuroprotection and enhance recovery after stroke.

## Effect of acupuncture on CNS

### Association between acupuncture sessions and its effects on motor function of Parkinson's disease

In their study, Lei et al. aimed to investigate the relationship between acupuncture sessions and the impact on motor function in individuals with PD. The researchers discovered that for patients with motor symptoms of PD, the effectiveness of acupuncture treatment may be influenced by the dosage administered. The study revealed that acupuncture with a specific dose was associated with better therapeutic effects. However, it is important to note that excessive acupuncture stimulation could lead to the development of tolerance within the body.

### Acupuncture manipulations impact blood pressure

The study conducted by Li Y.-J. et al. showed that acupuncture manipulations can effectively lower blood pressure. Among the different acupuncture techniques tested, it was found that twirling reducing manipulation had a stronger hypotensive effect on spontaneously hypertensive rats compared to twirling uniform reinforcing reducing and twirling reinforcing manipulations. Further investigations revealed that the anti-hypertensive effect of twirling reinforcing and reducing manipulation is likely associated with the activation of specific brain regions that play a role in regulating blood pressure. These brain regions are functionally connected and contribute to the overall hypotensive response. Interestingly, the activation of brain regions involved in motor control, cognition, and hearing was also observed during the acupuncture treatment. This suggests that in addition to regulating blood pressure, acupuncture may exert benefits on other aspects of brain function.

### Acupuncture for neurological sequelae of electric shock

Based on the information provided by Jiang, Zhang et al. a case study was conducted on a 29-year-old patient who had been receiving MA (most likely referring to medical assistance) for 17 months. The study focused on the patient's thalamencephalic and mesencephalic injury, which were found to be secondary to electrical trauma. The results of the study revealed that the patient's self-care ability and overall quality of life had experienced significant improvements. It can be inferred that the implementation of MA played a crucial role in enhancing the patient's ability to take care of themselves and led to a considerable enhancement in their overall wellbeing.

### Non-invasive techniques for migraines

In their study, Fernández-Hernando et al. examined the effectiveness of non-invasive neuromodulation techniques, specifically auricular transcutaneous vagus nerve stimulation and electro-ear acupuncture, in individuals suffering from migraine headaches. The researchers found that these non-invasive techniques showed some positive effects on the treatment of migraines, as reported in the current literature. However, it is important to note that the available data is still insufficient to draw definitive and strong conclusions regarding the effectiveness of these neuromodulation methods for migraine management.

### Acupuncture and Chinese medical herbs for tic disorders in children

Pu et al. conducted a thorough investigation that aimed to assess the quality and effectiveness of acupuncture as a treatment for TD in children. They analyzed and synthesized the data from several randomized controlled trials that have been published on this topic, with a keen focus on producing reliable evidence-based medical evidence. The study revealed that both acupuncture and traditional Chinese medical herbs have shown promising results in improving tic disorders in children. In fact, the researchers strongly suggested that these interventions may be the most effective therapies available for the management of TD in this specific population. Furthermore, when comparing the outcomes of acupuncture and traditional Chinese medical herbs with the commonly used Western medicine in clinical practice, the results clearly indicate that acupuncture, especially when combined with *tuina* therapy (a form of Chinese therapeutic massage), displayed superior effects in alleviating tic disorders in children.

### Acupuncture on prognosis and levels of brain-derived neurotrophic factor in acute phase of intracerebral hemorrhage

Li L. et al. conducted a research study aiming to explore the impact of acupuncture on the prognosis and levels of BDNF in patients experiencing ICH during the acute phase. The results of this study indicated that acupuncture, when administered in the acute phase of ICH, has a noteworthy positive influence on the short-term recovery of consciousness as well as long-term outcomes in patients with ICH. Furthermore, the production of BDNF may have a correlation with this beneficial effect observed through acupuncture treatment.

### Fire needle acupuncture for autoimmune encephalitis

In their study, Liu Y. et al. applied a technique called fire needle acupuncture to treat patients with autoimmune encephalitis, a condition that affects the central nervous system. The results of the study showed promising outcomes, indicating that the use of fire needle acupuncture led to positive effects and significant benefits in managing this autoimmune disorder. However, it is important to note that more convincing and robust data are still needed to further support the effectiveness of fire needle acupuncture as a treatment method for autoimmune encephalitis.

### Cheek acupuncture for severe neuralgia associated with peripheral nerve compression or permanent damage/dysfunction of the CNS

Wang Y. et al. reported two cases of patients who had symptoms of severe neuralgia associated with peripheral nerve compression or permanent damage/dysfunction of the CNS, and they introduced a cheek acupuncture. It can alleviate severe pain in patients with either peripheral nerve compression or permanent damage/dysfunction to the CNS, when analgesic medicines prove to be ineffective. The researchers observed an immediate and accurate analgesic effect in both cases after applying cheek acupuncture.

### Acupuncture for episodic migraine

Shi et al. sought to examine the long-lasting impact of acupuncture as a treatment for episodic migraine. The researchers found compelling evidence suggesting that the beneficial effects of acupuncture can persist for at least 3 months after the completion of the treatment. This means that individuals who received acupuncture experienced a significant reduction in the frequency and intensity of their migraine attacks for an extended period.

In summary, this Research Topic focuses on the exploration of noteworthy findings in the field of acupuncture from researchers around the globe. These findings specifically revolve around the utilization of acupuncture as a potential treatment for diseases associated with the CNS. This research comprehensively analyzed the profound impact of acupuncture on these CNS-related diseases. The researchers gained a thorough understanding of the therapeutic mechanisms through which acupuncture works, and subsequently unravel the intricate neural mechanisms that contribute to its overall effectiveness. Ultimately, the findings from this research may lay the groundwork for a theoretical framework, which can greatly contribute to enhancing the outcomes of acupuncture treatments for CNS diseases in clinical settings. This theoretical foundation may help equip researchers and healthcare professionals with valuable insights and knowledge that can further optimize the application of acupuncture in treating various CNS diseases, thus significantly improving the overall quality of patient care and treatment outcomes.

## Author contributions

QZ: Conceptualization, Data curation, Methodology, Resources, Validation, Visualization, Writing—original draft, Writing—review & editing. CL: Conceptualization, Data curation, Resources, Validation, Visualization, Writing—review & editing. XJ: Conceptualization, Data curation, Resources, Validation, Visualization, Writing—review & editing. HC: Resources, Validation, Visualization, Writing—review & editing. XL: Resources, Validation, Visualization, Writing—original draft, Writing—review & editing. JY: Resources, Validation, Visualization, Writing—original draft, Writing—review & editing. WP: Conceptualization, Data curation, Investigation, Project administration, Supervision, Validation, Visualization, Writing—original draft, Writing—review & editing. GY: Conceptualization, Data curation, Investigation, Project administration, Supervision, Validation, Visualization, Writing—original draft, Writing—review & editing.
